# Protective effect of KI in mtDNA in porcine thyroid: comparison with KIO_3_ and nDNA

**DOI:** 10.1007/s00394-014-0797-6

**Published:** 2014-11-09

**Authors:** Malgorzata Karbownik-Lewinska, Jan Stepniak, Magdalena Milczarek, Andrzej Lewinski

**Affiliations:** 1 Department of Oncological Endocrinology, Medical University of Lodz, 7/9 Zeligowski St., 90-752 Lodz, Poland; 2 Department of Endocrinology and Metabolic Diseases, Medical University of Lodz, 281/289 Rzgowska St., 93-338 Lodz, Poland

**Keywords:** Potassium iodide, Potassium iodate, Nuclear DNA, Mitochondrial DNA, Thyroid

## Abstract

**Purpose:**

Iodine, bivalent iron (Fe^2+^), and hydrogen peroxide (H_2_O_2_), all significantly affecting the red-ox balance, are required for thyroid hormone synthesis. Intracellular iodine excess (≥10^−3^ M) transiently blocks thyroid hormonogenesis (an adaptive mechanism called Wolff–Chaikoff effect). The aim of the study was to evaluate the effects of iodine, used as potassium iodide (KI) or potassium iodate (KIO_3_), in concentrations corresponding to those typical for Wolff–Chaikoff effect, on the level of oxidative damage to nuclear DNA (nDNA) and mitochondrial DNA (mtDNA) isolated from porcine thyroid under basal conditions and in the presence of Fenton reaction (Fe^2+^+H_2_O_2_ → Fe^3+^+^·^OH + OH^−^) substrates.

**Methods:**

Thyroid nDNA and mtDNA were incubated in the presence of either KI or KIO_3_ (2.5–50 mM), without/with FeSO_4_ (30 µM) + H_2_O_2_ (0.5 mM). Index of DNA damage, i.e., 8-oxo-7,8-dihydro-2′-deoxyguanosine, was measured by HPLC.

**Results:**

Neither KI nor KIO_3_ increased the basal level of 8-oxodG in both nDNA and mtDNA. KI—in all used concentrations—completely prevented the damaging effect of Fenton reaction substrates in mtDNA, and it partially prevented this damage in nDNA. KIO_3_ partially prevented Fe^2+^+H_2_O_2_-induced oxidative damage in both DNA only in its highest used concentrations (≥25 mM).

**Conclusions:**

Without additional prooxidative abuse, both iodine compounds, i.e., KI and KIO_3_, seem to be safe in terms of their potential oxidative damage to DNA in the thyroid. The superiority of KI over KIO_3_ relies on its stronger protective effects against oxidative damage to mtDNA, which constitutes an argument for its preferential utility in iodine prophylaxis.

## Introduction

All biological macromolecules are susceptible to oxidative damage, however, to a different extent. For example, nuclear DNA (nDNA) was more susceptive than membrane lipids to Fenton reaction (i.e., Fe^2+^+H_2_O_2_ → Fe^3+^+^·^OH + OH^−^) substrates in porcine thyroid [1]. In turn, the level of DNA oxidative damage in porcine thyroid was found to be approximately 10 times higher in mitochondrial DNA (mtDNA) [2] than in nDNA [1] in physiological conditions.

Among all DNA bases, 2′-deoxyguanosine bases are hot spots for DNA oxidative damage. The most important lesion deriving from this oxidation, and most frequently being examined, is 8-oxo-7,8-dihydro-2′-deoxyguanosine (8-oxodG).

Iodine is an essential trace element, which is indispensable for thyroid hormone synthesis. On the other hand, iodide excess, with intracellular concentrations exceeding 10^−3^ M, blocks this process [3]. This shutdown of thyroid hormone synthesis, called Wolff–Chaikoff effect, is a kind of adaptive mechanism, and it is transient [3]. Concentrations of iodine within thyrocytes as high as those typical for Wolff–Chaikoff effect may be potentially toxic for intracellular macromolecules, such as DNA, lipids, and proteins. However, such a hypothesis should be experimentally proven, the issue which is a subject of the present study.

The exposure of the thyroid to high doses of iodine may be caused by several factors, such as iodine containing drugs, contrast agents or, less frequently, seaweed preparations, or even iodized salt consumed in uncontrolled amounts, but the last one does not occur with ingestion of iodized salt under typical condition [3].

The only natural source of iodine is the diet. Due to frequently occurring iodine deficiency, precisely elaborated programs of iodine prophylaxis were introduced in different countries [4]. Iodine prophylaxis is based most frequently on obligatory salt iodization with the use of either potassium iodide (KI) or potassium iodate (KIO_3_).

Both the above compounds reveal similar effectiveness in iodine prophylaxis; therefore, they both have been proven to be used for fortifying salt. However, they differ in terms of certain chemical properties and potential toxicity. Potassium iodate is more stable, as iodide is readily oxidized to iodine and lost by evaporation [5]. However, human iodine bioavailability from KI is higher than from KIO_3_ [6], and in biofortification of vegetables with iodine, KI was found to be much more effective than KIO_3_ [7]. Also iodine concentration in cow milk was found to differ depending on iodide or iodate use [8].

It should be mentioned that some health authorities questioned the safety of KIO_3_ to humans and animals [9]. Iodine has been well known for its anti- and/or prooxidative properties. It is even hypothesized that dietary iodine is directly involved in the regulation of oxidative status in human breast milk [10].

The aim of the study was to evaluate the effects of iodine, used as KI or KIO_3_, in concentrations corresponding to those typical for Wolff–Chaikoff effect, on oxidative damage to nDNA or mtDNA in porcine thyroid under basal conditions and under conditions of experimentally induced oxidative stress by Fenton reaction substrates. Fenton reaction (Fe^2+^+H_2_O_2_ → Fe^3+^+^·^OH + OH^−^) is of special significance in the thyroid gland, as both its substrates, i.e., H_2_O_2_ and Fe^2+^, are required for thyroid hormone synthesis [11]. At the same time, Fenton reaction substrates are frequently used (also by authors of the present study) to experimentally induce oxidative damage to macromolecules in different tissues [reviewed in 12], the thyroid gland included [1, 2, 13–15].

## Materials and methods

### Chemicals

Potassium iodide (KI), potassium iodate (KIO_3_), ferrous sulfate (FeSO_4_), hydrogen peroxide (H_2_O_2_), alkaline phosphatase, and nuclease P_1_ were purchased from Sigma (St. Louis, MO, USA). MilliQ-purified H_2_O was used for preparing all solutions. All the used chemicals were of analytical grade and came from commercial sources.

### Animals

Porcine thyroids were collected from one hundred ninety two (192) animals at a slaughter-house, frozen on solid CO_2_ and stored at −80 °C until assay.

### Nuclear DNA isolation

Nuclear DNA was isolated and purified using a phenol extraction method [16] with some modifications introduced by authors of the present study, described before [1].

### Miochondrial DNA isolation

Mitochondrial DNA was isolated using an alkaline lysis method [17] with some modifications introduced by authors of the present study, described before [2].

### Nuclear and mitochondrial DNA incubation

Nuclear or mitochondrial DNA was incubated in 10 mM potassium phosphate buffer (pH 7.4) at a final volume of 0.5 ml in the presence of either KI or KIO_3_ (50; 25; 10; 5.0; 2.5 mM) without or with addition of Fenton reaction substrates, i.e., FeSO_4_ (30 µM) + H_2_O_2_ (0.5 mM). The concentrations of Fenton reaction substrates were experimentally established by us before [1, 2]. The concentrations of KI and KIO_3_ were selected on the basis of the results of the pilot study showing that in concentrations 0.01 mM or higher none of these compounds has changed the level of oxidative damage to nDNA or mtDNA. Additionally, the study aims are to examine the effects of high iodine concentrations corresponding to those typical for Wolff–Chaikoff effect. The incubation was carried out in a water bath at 37 °C for 1 h. For each type of DNA, three independent experiments were performed, and in each experiment DNA was isolated from sixteen (16) different thyroid glands.

### Evaluation of the 8-oxo-7,8-dihydro-2′deoxyguanosine/2′-deoxyguanosine (8-oxodG/dG) ratio

Evaluation of the 8-oxo-7,8-dihydro-2′deoxyguanosine/2′-deoxyguanosine (8-oxodG/dG) ratio was performed, as described before [1, 2].

### Statistical analyses

Results are expressed as mean ± SE. Data were statistically analyzed, using a one-way analysis of variance (ANOVA), followed by the Student–Newman–Keuls’ test. For respective concentrations of KI or KIO_3_, an unpaired Student’s *t* test was used. The level of *p* < 0.05 was accepted as statistically significant.

## Results

**Fig. 1:**
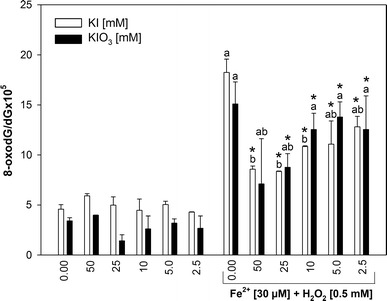
Oxidative damage to nuclear DNA in porcine thyroid. nDNA was incubated in the presence of KI or KIO_3_ [50; 25; 10; 5.0; 2.5 mM] alone or together with Fenton reaction substrates, i.e., FeSO_4_ [30 μM] plus H_2_O_2_ [0.5 mM]. Data are expressed as the ratio 8-oxodG/dG × 10^5^. Data are from three independent experiments. Values are expressed as mean ± SE (*error bars*). ^a^ *p* < 0.05 vs. control; ^b^ *p* < 0.05 vs. Fe^2+^+H_2_O_2_ (in the absence of KI or KIO_3_); *p < 0.05 vs. respective concentration of KI or KIO_3_ alone (i.e., in the absence of Fe^2+^+H_2_O_2_). Statistical evaluation was performed separately for KI (*white bars*) and for KIO_3_ (*black bars*)

Under basal conditions, two examined substances, i.e., KI and KIO_3_, did reveal similar effects, but in the presence of Fenton reaction substrates, effects of KI and KIO_3_ on oxidative damage to nDNA and to mtDNA isolated from porcine thyroid tissue differed substantially. Neither KI nor KIO_3_ did increase the level of oxidative damage to nDNA (Fig. 1).

**Fig. 2:**
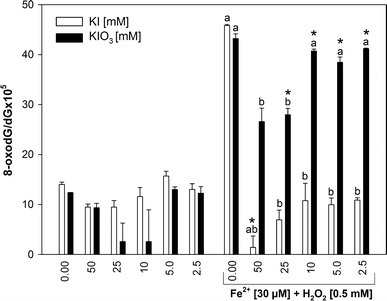
Oxidative damage to mitochondrial DNA in porcine thyroid. mtDNA was incubated in the presence of KI or KIO_3_ [50; 25; 10; 5.0; 2.5 mM] alone or together with Fenton reaction substrates, i.e., FeSO_4_ [30 μM] plus H_2_O_2_ [0.5 mM]. Data are expressed as the ratio 8-oxodG/dGx10^5^. Data are from three independent experiments. Values are expressed as mean ± SE (*error bars*). ^a^ *p* < 0.05 vs. control; ^b^ *p* < 0.05 vs. Fe^2+^+H_2_O_2_ (in the absence of KI or KIO_3_); *p < 0.05 vs. respective concentration of KI or KIO_3_ alone (i.e., in the absence of Fe^2+^+H_2_O_2_). Statistical evaluation was performed separately for KI (*white bars*) and for KIO_3_ (*black bars*)

When KI or KIO_3_ were used together with Fenton reaction substrates, both of them revealed concentration-dependent protective effects. Namely, KI in all used concentrations (50–2.5 mM) decreased Fenton reaction-induced oxidative damage to nDNA, with strongest effects observed for the highest KI concentrations (50, 25, and 10) (Fig. 1). In turn, KIO_3_ decreased Fe^2+^+H_2_O_2_–induced nDNA damage but only when used in the highest concentrations of 50 and 25 mM (Fig. 1). In case of mtDNA, neither KI nor KIO_3_ did increase the level of oxidative damage (Fig. 2).

In turn, when KI was used together with Fe^2+^+H_2_O_2_, it completely prevented—in all used concentrations—the damaging effect of Fenton reaction substrates to mtDNA (Fig. 2). Concerning KIO_3_, it decreased Fe^2+^+H_2_O_2_-induced oxidative damage to mtDNA only in its highest used concentrations, i.e., 50 and 25 mM (Fig. 2).

When effects of respective concentrations of iodine compounds were compared in the presence and in the absence of Fenton reaction substrates, the following results were found. In nDNA, the level of 8-oxodG/dG in the presence of KI or KIO_3_ (for most of concentrations) plus Fenton reaction substrates was significantly higher than in the presence of iodine compounds applied alone (Fig. 1). In case of mtDNA, these comparative analyses were completely different. Namely, the level of 8-oxodG/dG in the presence of KI plus Fenton reaction substrates was not higher than in the presence of KI alone (Fig. 2). Concerning the effect of KIO_3_, the level of 8-oxodG/dG in the presence of this iodine compound plus Fenton reaction substrates was significantly higher (for most of used concentrations) than in the presence of KIO_3_ alone (Fig. 2).

## Discussion

The first issue which should be discussed is to what extent iodine concentrations used in the present study correspond to physiological/pathological concentrations. Inorganic iodine concentration in the whole human thyroid is approximately 9 mM [18]. However, the intracellular concentration of iodine in human (and possibly in porcine) thyroid is estimated at the level from 20 to 500 µM [19] to even above 2 mM [20]. The lowest concentration of KI used in the present study, i.e., 2.5 mM, corresponds to the same order of magnitude of iodine concentrations causing Wolff–Chaikoff effect, i.e., 10^−3^ M (1 mM).

Only few studies have been performed till now to compare the effects of KI and KIO_3_. No differences were found between iodine content in different tissues or blood thyroid hormone concentrations [6] or lipid peroxidation level in the liver and the muscle [21] after in vivo treatment with high doses of KI or KIO_3_.

The present study is the second one to compare the effects of KI and KIO_3_ on oxidative damage to macromolecules in the thyroid. We have found before that KI prevented experimentally induced oxidative damage to membrane lipids in porcine thyroid; at the same time, KIO_3_ did not reveal any direct beneficial effects and even it caused strong prooxidative action on this process [13].

It should be mentioned that—due to very high molecular mass of iodine—molecular masses of KI and KIO_3_ are of the same order of magnitude. Thus, applied concentrations of KI and of KIO_3_ may be used to compare either the effects of iodide ions (I^−^) (formed from KI or KIO_3_) or effects of the whole compounds.

The fact that neither KI nor KIO_3_ did increase the basal level of 8-oxodG in both nDNA and mtDNA suggests that under basal conditions, i.e., without additional prooxidative abuse, both iodine compounds, producing intracellular iodine concentrations typical for Wolff–Chaikoff effect, are absolutely safe for thyroid DNA. Thus, iodine excess does not seem to create very dangerous conditions, at least in terms of its influence on oxidative damage to DNA.

In turn, in the presence of Fenton reaction substrates, effects of KI and KIO_3_ differed substantially and depended on the kind of DNA. Whereas 2.5 mM concentration, in which KI was protective to nDNA, reflects conditions corresponding with Wolff–Chaikoff effect, it is not sure if concentrations ≥25 mM, in which KIO_3_ was protective to nDNA, are reached in the thyroid intracellulary.

The superiority of KI over KIO_3_ was especially distinct in case of mtDNA. Whereas KIO_3_ in concentrations of ≥25 mM only partially prevented Fenton reaction-induced damage to mtDNA, protective effect of KI was spectacular as it was observed for all used concentrations, and the protection was complete. Therefore, our results suggest that KI approaching thyroidal mtDNA in concentrations typical for Wolff–Chaikoff effect may prevent oxidative damage to this molecule caused by coexisting prooxidative agents.

Potential mechanisms of differences between KI and KIO_3_ effects and between susceptibilities of nDNA and mtDNA to iodine compounds, observed in the present study, should be discussed.

First, whereas I^−^ acts almost directly (it requires only one step of oxidation to I_2_), IO_3_^−^ requires to be first reduced to I^−^ and the reduction of IO_3_^−^ requires the time and energy, and possibly, it is associated with unfavorable oxidative reactions and damaging effects. This difference between I^−^ and IO_3_^−^ constitutes the base for further following explanations.

Second, inorganic iodine neutralizes H_2_O_2_ in a two-step process, thereby preventing it from becoming a hydroxyl radical (^·^OH) in Fenton reaction pathway [22], thus blocking the mechanism of oxidative damage to macromolecules applied in the present study.

Third, it is postulated that antineoplastic effect of I^−^ is mediated by direct antioxidant/oxidant effects at the mitochondrial level [23]. Similarly, it is suggested that antioxidative/oxidative response of thyroid mitochondria to iodide excess contributes substantially to Wolff–Chaikoff effect [24]. Consistently, antioxidative mechanisms are evolved in mitochondria much better than in other cellular components [25].

Next, mitochondria are characterized by fast elimination of 8-oxodG [26], the product which is dangerous by itself [27], and this process can be enhanced by iodine compounds [28].

When discussing the possible direct effects of iodine compounds, it should be mentioned that KIO_3_ belongs to halogenate salts, which are known for their potentially toxic effects [5]. In our earlier studies, one of the halogenate salts, namely potassium bromate (KBrO_3_), being classified as a carcinogen, was shown in vitro (5 mM) and in vivo to exert damaging effect to membrane lipids in porcine thyroid [29]. Similarly, KIO_3_ increased lipid peroxidation in porcine thyroid homogenates when used in concentrations of 2.5–200 mM [13]. At the same time, KI (in concentrations ≤25 mM) did not reveal any toxic effects to membrane lipids and even it was protective in the broad concentration range [13]. Fortunately, iodate is characterized by the lowest redox potential among three halogenate salts, i.e., iodate, bromate, and chlorate; thus, it seems to be least toxic.

Summarizing, under conditions without additional prooxidative abuse, both iodine compounds, i.e., KI and KIO_3_, seem to be absolutely safe in terms of their potential oxidative damage to thyroid DNA. The superiority of KI over KIO_3_ relies on its spectacular protective effects against oxidative damage to mtDNA. This constitutes an argument for its preferential utility in iodine prophylaxis, relying either on salt iodization or on the use of tablets containing iodine, the latter recommended mainly during preconception, pregnancy, and lactation.

It should be also stressed that oxidative damage to mtDNA in the thyroid (as well as in any other tissue) in response to iodine has never been studied before, and our findings are promising in terms of prevention of iodine deficiency disorders, especially cancer.
